# Protein Microarrays with Novel Microfluidic Methods: Current Advances

**DOI:** 10.3390/microarrays3030180

**Published:** 2014-07-01

**Authors:** Chandra K. Dixit, Gerson R. Aguirre

**Affiliations:** Biomedical Diagnostics Institute, Dublin City University, Glasnevin, Dublin-9, Ireland; E-Mail: gerson.aguirre@dcu.ie

**Keywords:** microarray, micromosaic, microfluidic networks, centrifugal arrays, microbeads

## Abstract

Microfluidic-based micromosaic technology has allowed the pattering of recognition elements in restricted micrometer scale areas with high precision. This controlled patterning enabled the development of highly multiplexed arrays multiple analyte detection. This arraying technology was first introduced in the beginning of 2001 and holds tremendous potential to revolutionize microarray development and analyte detection. Later, several microfluidic methods were developed for microarray application. In this review we discuss these novel methods and approaches which leverage the property of microfluidic technologies to significantly improve various physical aspects of microarray technology, such as enhanced imprinting homogeneity, stability of the immobilized biomolecules, decreasing assay times, and reduction of the costs and of the bulky instrumentation.

## 1. Introduction

Disease diagnostics is performed by immunoassays developed against a specific analyte/antigen. Immunoassays for such applications are developed using an established technology, which is based upon microtitre well plates [1]. Microarray is another important technology in the field of diagnostics, especially in genetic analysis. Microarrays are defined by a matrix of several individual recognition elements immobilized on a solid support (substrate) [2] using bulk-phase mass transfer strategy, in particular by direct spotting of biomolecules on the substrate. The major benefits of microarray over microtitre-based immunoassay are multiplexity (a couple thousand spots in low density array to 50,000 spots in high density arrays), low sample volumes, robustness, and rapid methods. At the same time, the major drawback associate to microarrays is data analysis that requires complex algorithms and extensive computation. 

**Figure 1:**
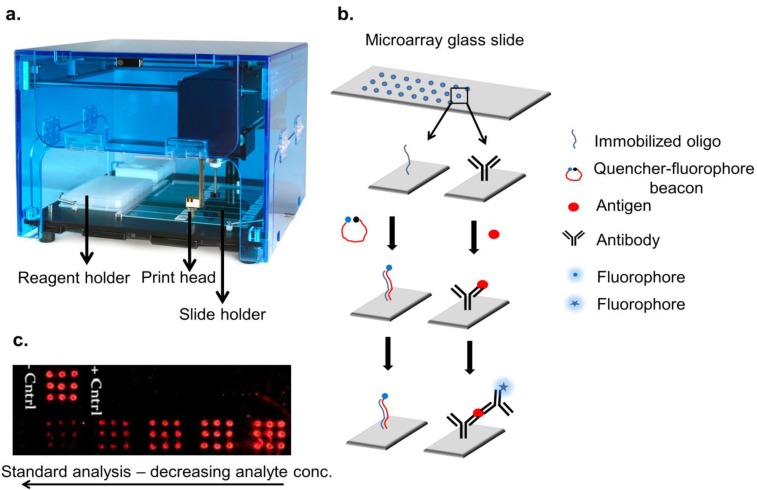
Illustration of microarray technology. An array printer is depicted in (**a**) which has a dedicated space for holding reagent plates and slides. An automated syringe cum pin is filled up with specific reagent followed by spotting in specific spot-sizes and electronically controlled volumes (Table 2); (**b**) Methodology of creating oligo/antibody array is depicted. Each spot on the slide holds immobilized oligonucleotides or antibodies which were later used for analyte detection; (**c**) An assay in a microarray format is shown with immobilized anti-fetuin antibody detecting various concentrations of fetuin and detection performed with Cy5 anti-fetuin antibody. Spot size of the array is 2 mm each, with a spotting volume of 1 µL anti-fetuin capture antibody.

There are several methods for developing microarrays that include printing/spotting, *in situ* synthesizing on solid supports, high-density and suspension bead arrays, and electron microarrays are few of the examples [3]. The nature and types of recognition elements are used to categorize the type of microarray. The most common recognition elements are nucleic acid and proteins that are spotted/printed on the substrate; there are several other recognition elements, such as microRNA-, peptides-, and glycans (Figure 1; Table 1). Microarrays have demonstrated tremendous potential in the field of diagnostics because they enable low-volume multiplexing analysis. The two key fields associated to the microarray development are genomics and proteomics; however, the advances in microarray technology contributed significantly in progressing genomics, in comparison to proteomics [4]. Today, high-density DNA arrays are available for the detection of genes with a respectable number of the commercial products having been approved by the food and drug administration (FDA), USA [5]; however, key to the fulfillment of clinical diagnosis potential is based on protein-based biomarkers. Gene-based diagnostics can only provide information on the genetic susceptibility of a person towards a disease but generally gene expression is not representative of the amount and nature of protein present in/outside the cells [6], which constitutes the major problem for using DNA-based diagnostics. Genomic encoding applications have been developed for cancer management and analyzing the treatment course of patients, such as determination of chemotherapies for cancer treatment and serial analysis of gene expression (SAGE), have been possible due to the high-parallel processing afforded by microarrays explicitly. Protein microarrays have now become common in research and development industries in the form of antibody arrays, which are employed as analytical and diagnostics tools [7]. However, their use still has not become routine in clinical diagnostics due to numerous reasons. The most obvious is complex analysis of obtained results in addition to high cost of development and intensive labor requirements [8]. Additionally, major challenges are validation of the developed microarrays in terms of the analyte detection ability at variable concentrations and reproducibility of the data for the same set of samples over an extended period [9,10]. Meeting strict regulatory norms in the US and Europe for public use of various *in-vitro* diagnostics (IVD) platforms has further complicated the inception of protein microarrays as a routine clinical diagnostic tool [11]. The lack of exhaustive testing of protein microarray platforms and the restricted availability of information regarding proteins and their expression in public domain adds further to the hurdles in their use for clinical set-ups. Automation, robustness, and reliability of these microarray platforms are some of the most critical parameters that have not been completely overcome, which have prevented major diagnostic companies to enter into the microarray-based IVD market. The investment and achievements therefore have resulted in only few microarrays for specific diseases now commercially available.

Microarray technology has benefitted the scientific and clinical community by enabling quick, low-volume analysis of samples. However, like microtitre plate-based methods, microarray methods are bulk-dependent and are diffusion-limited [4]. In the past few years, new microarray development technologies were developed by the introduction of microfluidics-based approaches. This has enabled us to address previous drawbacks associated with microarrays by leveraging fluid displacement properties at the micron scale, a high surface area to volume ratio, which accelerates the diffusion-limited processes [5], and their compatibility with disposable materials for rapid prototyping [12]. Recently, microtitre plate-based immunoassay technology has also seen substantial improvements especially its merger with microfluidics-leveraged processes [13,14] but the main focus of this manuscript is to review techniques and methods for developing microfluidic microarrays. On one hand, while microfluidic microarrays provide rapid, robust methodological alternatives, on the other hand, a major challenge of this hybrid technology will be the development of high-density arrays that is an issue, quite well addressed in conventional methods but have required sizable investment. On the other hand researchers have devised a new convenient microfluidics-based arraying method that has been demonstrated at research level as a potential replacement for the current technology. The concept of developing array patterns using a microfluidic network (µFN) system, known as *micromosaic*, was first introduced in 2001 by Delamarche and his group in the Life Science division at IBM-Zurich, which was later adapted by many research groups [15]. Other microfluidic platforms that were developed for microarray applications are microbead-based and centrifugal system-based. Successful attempts have also been made to merge conventional microarray technology with advanced microfluidic-based reagent delivery systems [16]. In these approaches, recognition elements or probes are first spotted on the surface using conventional methods then a microfluidic cartridge is aligned on the spotted chip for delivering analytes over those spots. Microfluidics has thus provided an edge over conventional approaches of microarray development. The simplicity of this method is very cost effective, even with the use of microfluidic systems. 

**Table 1:** Different microarray types employed for disease diagnosis.

Type of microarray	Method of development	Format	Density	Diagnostics application	References
DNA	Printing, *in situ* synthesis	Oligonucleotide, cDNA	Low-High	Respiratory, digestive tract infections	[17,18,19,20,21]
RNA	Printing	miRNA, total RNA	Low-High	Liver diseases, viral miRNA	[22,23,24,25]
Protein and Peptide	Printing, stamping	Immunoassays, enzymatic assays, label-free	Moderate	Biomarker discovery, bacterial antigen	[1,14,26,27,28,29,30,31,32,33]
Carbohydrate	Stamping, Drop-coating	Lectin assay	Moderate	Cell signaling, Biomarker discovery	[34,35,36,37]
Cellular	Droplet-coating	Immunoassays, protein assays, molecular assays	Low-moderate	Biomarker discovery, drug discovery, CTC identification	[38,39,40,41]

## 2. Development of a Microarray

This is a complex process that involves array fabrication (solid support selection, functionalization and immobilization of recognition element), assay development, and device optimization. It is critical to pack high density number of recognition elements on the array surface, about 1 cm^2^, in order to achieve highly parallelized throughput sample analysis, which is important specifically for gene-level diagnostics and proteome research. Most common techniques for the development of microarrays are contact and non-contact printing and have been summarized in Table 2. 

**Table 2:** Various conventional microarray fabrication methods.

Method	Principle/Description	Print density	Relative cost	References
**Contact Printing**	Reagent spotting on the desired surface either with a thin capillary-like nozzle/tip or with conformal contact of a biomolecule-coated stamp			
Pin-based	A nozzle dispenses solution with a print head; dispensable volume varies (0.5–5 µL); spot sizes varies between 20–200 µm in diameter	Moderate	**$$**	[42,43,44]
Nanotip-based	An AFM tip dispenses solution onto the surface; dispensable volume varies (0.1–0.3 µL); spot size varies between 30–100 nm in diameter	High	**$$$**	[45,46]
Microstamping	A polymer cast with specific spot-patterns is dipped in desired protein solution that can replicate these protein spots on any surface by conformal contact	High	**$**	[47,48,49,50]
**Non-contact Printing**	Spotting is performed without making a conformal contact with the surface; reagents are delivered either by spraying or localizing under the influence of various fields			
Inkjet-based	A nozzle sprays the 4–8 nL volume of the reagent as a liquid jet; spot size is variable and poor resolution	Moderate	**$**	[51,52,53,54,55,56,57]
Laser writing	Laser ablation of the thin film generates spatially localized evaporation that creates a confined droplet of sample/reagent placed parallel to the ablation zone; spot size 10–100 µm in diameter.	Moderate	**$**	[58,59,60]

Selection of solid supports used for developing these microarrays is critical in terms of achieving homogeneous spots of specific sizes. Hydrophobic surfaces have a high tendency towards adsorption of proteins and are regularly used for developing immunoassays in ELISA format; however for microarray development hydrophobic surface results in a non-homogeneous protein distribution [61]. Therefore, several modified surfaces have been reported for development of microarrays. Most commonly employed supports are epoxy-/aldehyde-/amine-activated glass surfaces, plastics functionalized with silanes or layer-by-layer assemblies, and polymeric materials and are summarized in Table 3. 

**Table 3:** Solid supports, their chemical nature, and grafted functionalities for microarray development.

Solid Support	Inherent Chemical Nature	Functionalization Strategies
**Plastic polymers**		
Polystyrene	Hydrophobic	**For functionalizing surfaces:** • Step 1: Activation of the surface with oxidation (chemical or plasma method) • Step 2: Incubation with silanes (amine/carboxy/epoxy) or with poly-L-lysine • Step 3: Further functionalization with dendrimers for achieving stability of the silanes on the surface **For enhancing anti-fouling properties of hydrophobic surfaces:** • Activation of the surface with oxidation (chemical or plasma method), or • Incubation with silanes (amine/carboxy/epoxy) or with poly-L-lysine, or • Coating with surfactants, such as tritonX-100, tween-20 and polyvinyl pyrrolidone
Polymethyl methacrylate	Hydrophobic	
Poly carbonate	Hydrophobic	
Cyclic poly-olefin	Hydrophobic	
Cellulose acetate	Hydrophilic	
**Non-plastic polymers**		
Glass	Hydrophilic	
OSTE	Hydrophilic	
PDMS	Hydrophobic	

Another most important aspect in microarray development is immobilization of the recognition elements, such as antibodies or nucleic acids. Covalent, site-specific, random, and oriented immobilization are the most common approaches for depositing biomolecules on surfaces (Table 4). On the epoxy-/aldehyde-/amine-activated surfaces proteins are mainly coated via covalent linkage [62]. Additionally, recognition molecules can be physio/chemisorbed on the support material via hydrophobic, electrostatic and/or co-ordination chemistry [62,63,64]. Covalent and adsorption based methods account for the random capture of proteins because appropriate presentation of active site of these molecules over the surface is not controlled. This could lead to a highly heterogeneous surface where a significant proportion of these recognition molecules may lose their activity due to the interaction of their active sites with surface [65]. In order to address this drawback researchers have employed site-directed and oriented immobilization methods by using a precapture stage which facilitates an orderly presentation of the functional sites of the recognition molecules [66]. In site-specific protein capture methods one molecule of the affinity pair is introduced on the protein while surface is functionalized with the other member of the affinity pair thus guiding the immobilization to a specific site of the protein. Examples of such tags are streptavidin-biotin or FLAG tag, and enzyme-mediated substrate reactions [62,63,64]. Other precapture proteins widely employed for oriented immobilization are antibodies, anti-antibodies, and staphylococcal proteins *viz*. protein A, G, or AG [62].

**Table 4:** Protein immobilization methods for microarray applications.

Attachment Method	Nature of Surface	Mechanism of Binding	Treatment for Binding	References
**Adsorption (physisorption)**	Hydrophobic (*high density*) Hydrophilic (*low density*)	Interaction of hydrophobic pockets with that of the surface van der Waals interactions Hydrogen bonding	Proteins incubated at basic pH, such as carbonate-bicarbonate buffer pH 9.2 for exposing buried hydrophobic pockets	[62,67]
**Adsorption (Chemisorption)**	Ionic bonds Coordination bonds	Interaction of ionic species Interaction of chemical species of protein and surface via non-covalent dative bonds	Treatment with acidic or basic buffer for rendering amines or carboxyls charged Exposing sulfhydryls for reaction with metallic surface	[62,67,68]
**Covalent, random**	Grafted with pendent amine, carboxyl, sulfhydryl, epoxy, and other functionalities	Covalent bond between amine, carboxyl, and hydroxyl of proteins with those of surface	Mediated by crosslinkers such as NHS esters, carbodiimide, glutaraldehyde	[62,63,64,67,68]
**Covalent, site directed**	Grafted with pendent amine, sulfhydryl, and carboxyl functionalities	Covalent bond between hydroxyl, sulfhydryl, and aldehydes of the protein with those of the surface	Crosslinker mediated reaction via tosyl, tresyl for reaction of hydroxyls of protein Direct reaction for aldehyde of protein with amines of the surface Crosslinker mediated reaction of sulfhydryls via maleimide, pyridile, and haloacetyls	[62,63,64]
**Interaction-based oriented binding**	Biofunctionalized surface with streptavidin, antibody-binding protein A, G, AG or L, FLAG tag, Ni^+2^-NTA tag, Enzyme-substrate reaction-mediated	Van der Waals, hydrophobic interaction, hydrogen bonding Covalent bonding	No pretreatment required	[62,63,64,66,67,68]

In the last decade, facile, robust, quick and less labor intensive approaches have been developed in microarray technology. High-density bead array coupled with microfluidics is one such example where the entire application lies on the ability to spatially decode the identification of each individual bead with the benefit of larger surface area per unit bead over that of planar arrays for binding which provides an advantage when considering the density number of probes available for binding. Microfluidic network-based microarrays, simply known as micromosaic, are yet another example that has been developed as a demonstration of the technology. 

## 3. Microfluidic Networks (µFN)

### 3.1. Micromosaics

**Figure 2:**
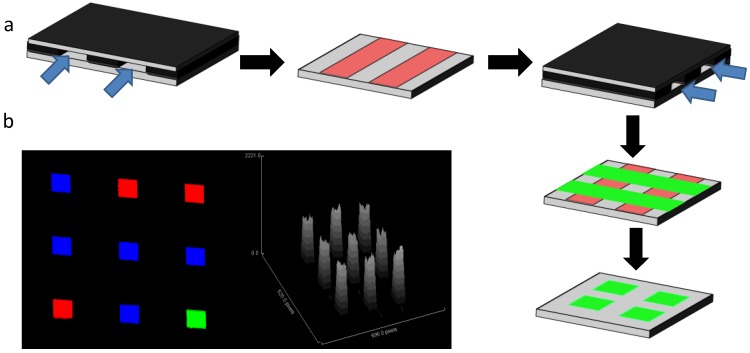
Methodology and results for developing micromosaic microarray using µFNs. Flow of biorecognition element of choice in the first direction using a network of parallel channels (**a.i**); immobilized strips of the biomolecule are created (**a.ii**); secondary analyte solution is flown over the immobilized biomolecule strips at 90 °C (**a.iii**); which creates second dimension lines (**a.iv**); washing steps remove the undesired portions of the strip leaving spots of the size of channels (**a.v**); A 3X3 micromosaic illustration is shown in (**b**) which was developed as an adaptation from a report by Bernard *et al*. [15]. We have employed a µFN of three parallel PDMS channels of 50 µm width, 20 µm depth, and 1 cm length treated with plasma for rendering channels hydrophilic thus allowing reagent delivery by capillary. Arraying was performed on a PDMS slab with anti-goat IgG-Cy3 and goat IgG-atto647. Red, blue, and green represent different fluorescence emission wavelengths, while bars are the intensity of each spot.

This is one of the most effective methods developed in recent times for microarray fabrication. The method can be described as arraying of recognition elements in one dimension in form of a stripe followed by flowing analyte on the surface in a perpendicular dimension (Figure 2). The cross-section of two stripes perpendicular to each other result in an area of reactive site equal to the widths of the used channels. This method is known as microfluidic network (µFN)-based array fabrication, and the resulting array is referred to as a micromosaic. The number of channels in a µFN governs the number of spots per chip, thus, a geometric growth dependency is formed. While steric and kinetic considerations exist, this technique allows the adequately accessibility of the immobilized probes to the target molecules from a surface energetics perspective [69]. Surfaces are important consideration when developing micromosaics. Polymeric substrate materials are inferior to glass substrate, however, both these solid supports require adequate functionalization for proper analyte capture via flowing within the channels, as well as prevention of non-specific binding leading to false positives. For efficient reagent loading, channels in µFN are made hydrophilic by various approaches, such as plasma exposure or by chemical treatments, which allows filling these channels with biomolecules using a combination of positive surface tension and capillary forces [70]. This simplifies the reagent delivery instrumentation and removes the need for robotic spotting. Micromosaic technology was reported for the first time by Bernard *et al*. [15] where they demonstrated this technology with a direct immunoassay by coating an antigen onto polydimethylsiloxane (PDMS) surface along the X-axis and fluorophore-labeled detection antibody along Y-axis. Thus, the number of these spots was controlled by the number of channels in X- and Y-direction. Increasing the number of microfluidic channels in both directions increases the number of spots. In their work they have always employed silicon surfaces with engraved channels functionalized with silanes and further grafted with hydrophilic coatings for capillary-based reagent delivery. Subsequently, several other research groups have employed this technology either in its original form or modified it according to their requirements.

Jiang *et al*. (2003) have integrated micromosaics and reagent dilution system for detecting HIV-related gp-41 and gp-120 proteins [71]. They patterned antibodies in the first dimension using regular microchannel system and integrated another micro-dilutor in the second dimension. Cesaro-Tadic *et al*. (2004) reported a modification by introducing an 11-channel capillary flow system [72]. Introduction of a capillary system allows a continuous flow of reagents thus omitting the requirement of pretreatment of the channels for making them hydrophilic. Plug-flow techniques in microfluidics were employed for significantly reducing the hybridization times [73]. This type of flow approaches has a natural recirculating mixing effect by displacing the reagent several times over the array probes with larger sample volumes inside a microfluidic confinement. In addition, Gao *et al*. (2005) reported the use of electrokinetic flow control for development of micromosaic technology [74]. In their design the coating of antigens was similar to a typical micromosaic; however, they employed an ‘H’-shape microfluidic assembly for allowing electroosmotic flow-mediated delivery of the analytes to the coated antigen. This modification in the original micromosaic design was to facilitate the current flow through an intersection channel for controlling the flow speed over the spotted antigens. Hunziker and colleagues (2007) employed a capillary driven µFN assembly for capturing cells and for performing surface receptor screening in micromosaic immunoassay format [75]. As a conceptual proof they have captured mouse hybridoma cells on an anti-CD44 antibody coated on PDMS surface. Takayama and colleagues (2007) replaced the use of thick PDMS block with membranes [76]. They sandwiched a semi-porous polyester membrane between glass and PDMS channels. They achieved a multilayer microfluidic system for analyzing the effect of various reagents on the protein expression conditions of C2C12 myoblast cells where reagents were flown continuously in the microfluidic channels and allowed to diffuse through polyester membrane. In addition, Liu *et al*. (2012) reported the development of mosaic patterns on filter membranes by sandwiching it between two layers of PDMS. Furthermore, they accelerated the binding of the probe on the array and detection via immunoassay by applying a vacuum [77]. Ziegler *et al*. (2008) reported a further modification to the initial design where they developed a stencil network for directing and coating proteins in several spots [78]. This modification had less fluidic components as the probe array deposition was replaced by stencil patterning. Shao *et al*. (2011) developed an array using a double-layered microchannel system [79]. Each layer in their system possesses a microfluidic network; the network in upper layer was perpendicular to the lower network thus developing a pattern similar to the micromosaic system. However, unlike the regular micromosaic patterning there were no removable parts in this chip, thus, eliminating the need of removing µFN of first dimension and reintroduction into the other. Most of the initial designs for micromosaics that researchers have followed are summarized in Table 5. In most of the micromosaic-based studies fluorescence, plasmon, and bioluminescence were commonly used for signal detection and analyte recognition.

### 3.2. Other Networks

Several µFNs were developed but most of them were employed for the development of hybridization-based microarrays. Bergeron and colleagues (2005) were few of the first to develop such a system [80]. Later, Kartalov *et al*. (2006) developed a complex µFN for arraying proteins with on-chip pneumatic valves for controlling the type of solutions and amounts in the microchannels [81]. They have demonstrated the detection of five different proteins in 10 different samples. Their group further improved the initial design of the µFN and increased the number of detectable proteins to a matrix of 6X10 [82]. Yu *et al*. (2009) developed a novel method for developing epoxy-functionalized microchannels and demonstrated its use in developing 3D protein microarray [83]. Recently, Roy *et al*. (2011) have employed the microfluidic network for the detection of miRNA detecting as low as 300 copy numbers [84]. This is an emerging field and continuously researchers are showing inclination toward this technology for developing microarrays. µFNs are highly advantageous because of the ease and high efficiency of patterning proteins on the surface and, thus, development of multi-spot microarrays. Several other physical process that significantly affects the protein spotting stage of the development process of spotting-based microarray include evaporation, incubation times, temperature, *etc*.; on the contrary, in microfluidic microarrays, the effects of such physical entities are negligible thus require minimum optimization. In addition, in microchannels the protein immobilization is faster due to the increased mass flow in comparison to the conventional spotting-based microarray methods. In addition, fabrication of µFNs is easy. Such fluidic networks are now regularly employed for point-of-care applications [85,86].

**Table 5:** Summary of the developed micromosaic immunoassays.

Detection Method	Solid Support	Assay Format	Analyte	Sensitivity	Spots/Array (n × n)	Reference
Fluorescence	Silicon	Direct immunoassay	Guinea pig IgG	6 ng/mL	25 × 25	[15]
Plasmon	PDMS	Hybridization	RNA/DNA			[87]
Fluorescence	Glass	Sandwich immunoassay	Bacteria		6 × 6	[88]
Fluorescence	PDMS	Direct immunoassay	Gp41; gp120			[72]
Fluorescence	PDMS	Sandwich immunoassay	Human TNF	20 pg/mL	10 × 17	[73]
Bioluminescence	PDMS	Intracellular signal	Cells		5 × 5	[89]
Fluorescence	PDMS	Sandwich immunoassay	C-reactive protein	30 ng/mL	7 × 7	[90]
Fluorescence	PDMS	Direct immunoassay	Multiple Bacterial antigens		1 × 5	[74]
Fluorescence	PDMS	Direct assay	Cells		8 × 6	[91]
Fluorescence	PDMS	Direct immunoassay	Antibodies against bacteria in serum		6 × 6	[92]
Fluorescence	Silicon nitride	Sandwich immunoassay	C-reactive protein	2.5 µg/mL	3 × 12	[93]
Fluorescence	PDMS	Quantum dot-based sandwich immunoassay	Carcinoma embryonic antigen	500 fM	4 × 8	[94]
Fluorescence	PDMS	Sandwich immunoassay	C-reactive protein	1ng/mL	5 × 14	[78]
Fluorescence	PDMS	Sandwich immunoassay	Oxidative stress biomarkers 3-nitro tyrosine, Catalase Superoxide dismutase	150 µM 5 ng/mL 0.5 ng/mL	3 × 10	[95]
Fluorescence	PVDF, PDMS	Direct immunoassay			1 × 10	[96]
Fluorescence	PDMS	Direct immunoassay	IgG	5 ng/mL	1 × 4	[79]
Various	Various		Various			[97] (Patent number: US 8,075,854 B2)
Fluorescence	Polycarbonate and PDMS	Sandwich immunoassay	Rabbit IgG	0.16 µM	6 × 5	[77]
Fluorescence	PDMS	Sandwich immunoassay	Panel of HIV associated antigens		8 × 21	[98]
Fluorescence	PDMS	Sandwich immunoassay	Panel of HIV associated antigens			[99]

**Figure 3:**
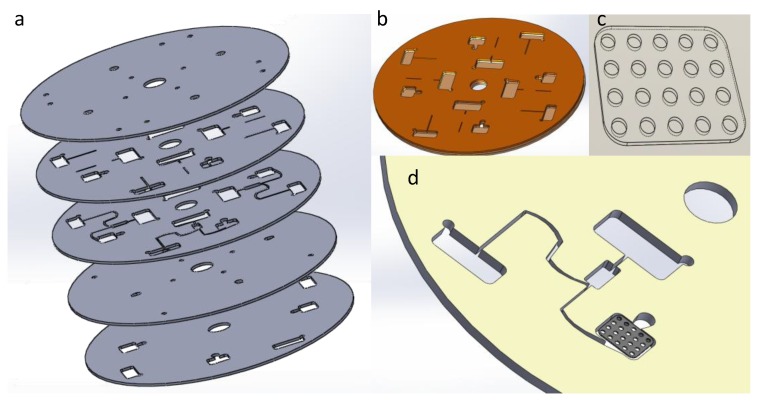
An illustration of the disc-based microfluidic microarray platform developed by Noroozi *et al*. [102]. (**a**) represents the burst-out of the whole platform where the main compartment as shown in (**b**) holds the arrayed paper (**c**) in the compartment; (**d**) shows the close up of the reagent delivery system to the array.

Centrifugal force driven microfluidics have had a great impact on flow control and development of diagnostic devices in the form of Lab-on-chip or –CD (developed on circular disk-like platforms) (Figure 3). It is also another method reported for the development of microfluidic microarrays. Liquid propulsion is generated by spin frequency and accelerations, which govern the fluid movement in the channels. Induced forces in centrifugal driven systems, such as Coriolis and physical volume forces, enhance mixing by accelerating the diffusional process [100]. In addition, susceptibility of centrifugal platforms to physiochemical properties of the fluids is negligible due to some of the typical materials used with pressure sensitive adhesives (PSA). Several DNA microarrays have been reported on this platform; however, restricted literature is available for protein patterning-based applications. The earliest of such microfluidic microarray was reported by Bynum and Gordon (2004) where they have integrated a spotted nucleic acid chip with a centrifugal platform, which uses concentric co-rotating chambers [101]. Noroozi *et al*. developed a microarray system for detecting IgG protein immunoassay and *Burkholderia* antigens printed on a nitro-cellulose membrane [102]. The device’s operational method relied on flow-reciprocation, which focused on re-flowing of analytes over the same surface area for increased absorption to reduce cost and time. 

## 4. Microbeads

**Figure 4:**
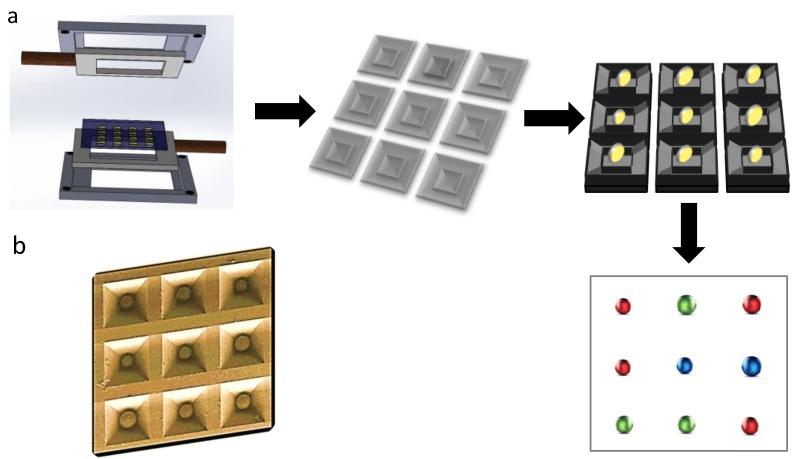
A set-up of the device is illustrated in (**a**) for creating a surface with several microwells. Later these microwells are filled with antibody-functionalized microbeads depicted in yellow colour such that each well holds one bead, which makes a micro-site for performing immunoassays. Detection is performed using fluorescence microscopy with appropriate emission filters. Red, green, and blue represents the fluorescence emission filters for respective fluorophores; (**b**) shows an array of microbeads adapted from [104].

Microbeads have now become popular due to their ease of handling, processing efficiency and ability to be used in wash-free immunoassay development. These beads are available in different sizes and are customizable in terms of their chemical functionalization and biomolecule immobilization. They have started to gain popularity in protein patterning as well. Several of such applications are already available in the market (Illumina, B&D, Affimetrix) (Figure 4). Probe-functionalized beads are generally trapped in microwells or microchambers and then bioassays are performed on them under flow conditions. Goodey *et al*. (2001) for the first time reported the development of a fluidic microbead array [103]. Specific receptor-labeled beads were filled in an array of microcavities followed by flowing analytes over it. They have demonstrated the use of microbead array for the detection of influenza virus in addition to the solid-phase peptide synthesis. In a similar approach, McDevitt and colleagues (2002) have developed an array for the detection of C-reactive protein (CRP) [104]. Ali *et al*. (2003) reported a microbead-based microarray [105], demonstrated where beads were employed for generating arrays They have generated a network of microtrenches of the dimensions of 400 microns in an inverted pyramid shape that can hold a bead under flow. These microtrenches were aligned over with a microfluidic network followed by flowing the analyte, thus, facilitating specific DNA fragment hybridization. Barbee *et al*. (2010) developed a microfluidic system where they have incorporated a bead capture module for capturing specific antibody-functionalized beads and an electrophoretic module underneath it for protein separation [106]. The separated proteins were allowed to react with electrophoretically separated proteins thus detecting them in an array pattern. Yu *et al*. (2010) also have employed microbead-based microfluidic arrays for detecting a panel of seven receptors viz. epidermal (EGFR), insulin-like 1 (IGF-1R) platelet-derived receptor beta (PGFR-beta), human epidermal receptor 2 (HER2), vascular endothelial receptor 2 (VEGFR 2), and tyrosine kinase, associated to growth factor-mediated signaling [107]. Yang and colleagues (2010) used a modified microbead array where they incorporated microcontainers to a µFN and employed it for high-sensitivity viral detection as low as 1000 copy numbers/mL [108]. Thus, microbead-based method provides a robust and facile approach to develop microarrays with an advantage of customization. This approach has a potential to revolutionize the way we perceive personalized diagnostics.

## 5. Perspective

Sequencing technologies available today for molecular analysis are providing vast avenues for the development of biological and clinical applications with next-generation-sequencing (NGS) leading the forefront. Microfluidic platforms are playing a crucial role in the advancement of state-of-the-art. However, these systems must phase with other advancing technologies in order for the innovation to continue. The landmark technological alternate to Sanger method of sequencing, sequencing-by-synthesis, was revolutionized by Church's group, which allows the arraying of molecular materials in picoliter volumes. Microarrays are the key to the cost effective success of genome analysis but their use in protein biology and analysis of circulating DNA and RNA is restricted. Since proteins are a better diagnostic tool than genetic approaches therefore, diagnostic devices will have to be developed with protein handling capabilities by overcoming denaturation issues; firstly in terms of their immobilization on surfaces [67,68] and secondly for long term storage [65]. Other issues also concern the read length to provide reference of sequencing or a directive from the genome *vs.* characterization of sample for novel identification of molecular material. Conventional methods for arraying are still preferred to develop microarrays due to some unmatched requirements related to automation and low-throughput-, as well as the loss of biomolecules due to non-specific binding, to which many solutions have been identified, but it remains specific to the type of material used [109]. These two obstacles confluence and created the need of the development of automation strategies, such as valving techniques, to create high-throughput analytics while reducing cross-contamination. NGS systems can provide same output of reads in 24 h as hundreds of Sanger-type capillary sequencers, however, to replace them, microarrays at present will not supersede this processing power, nor come anywhere close. Conversely, for reagent delivery electrokinetic systems, as demonstrated by Gao *et al*. (2005), are best suited at micron scales and allows automation of various microfluidic technologies [74]. Further automation, which currently is an engineering challenge, can be achieved by on chip incorporation of several other microfluidic modules, such as patient sample handling platforms and cell separation for screening parallelization, which have been exploited at functional level [110,111]. These integrations might well induce a market interest to invest and reap benefit as it is doing with the NGS development specifically into the Biochip.

Additional problems in the development of microfluidic microarrays are validation of the developed assays [112,113,114,115] and reuse of the chips [116]. The validation problems are attributed to the high variability in the imprinting due to the fact that patterning in two dimensions require subsequent removal of the microfluidic network of first dimension for introducing the network in second dimension; this restricts the PDMS bonding to the substrate only by conformal contact. Therefore, we need to depend upon self-propelled or flow displacement techniques, such as capillary pumping [117]. This introduces huge variability due to the mass-transport phenomena limitations in sensing and detection, which are complex and difficult to mathematically model due to the requirement of performing analysis at individual component levels. Thus, this needs an in-depth understanding of operational mechanism of the device. These theoretical developments help in designing biosensors in microfluidic devices which can be used to assist in understanding the complex phenomena happening in these systems therefore, advection flow regime must be dominant when designing such microfluidic microarrays in order to reduce such mass-transport limited variability. In addition, fine tuning of interfaced upstream components and a precise control over flow rates must be attentively addressed to ascertain transport phenomena regime. Developed immunoassays on these fluidic arrays can be validated by analyzing several rounds of assay performance on separate arraying units as well. This will allow finding out variations among repeats of the fluidic arraying technique and the performance of the assays. In addition, cross platform validation, such as against conventional arraying techniques, will be helpful in further analyzing the arraying efficiency by microfluidic methods. Other issues, such as calibration and use of reference samples have led to more consistent output and less intra-assay variation [113,114,118] especially due to the low aspect ratio of microfluidics. Additionally, speed of reactivity to provide information about affinity and cross-reactivity of the sample and low consumption of sample and reagents have positive benefits over conventional methods. Furthermore, the effects of contaminants and nutrient exchanges in the case of cell micromosaic arrays [75] as compared with larger volumes of cells need to be understood to maintain adequate response from sampled population. Permeability of molecules and gases and leachability of material are also a concern as, for example, ubiquitous PDMS has shown in some instances to have adverse effects on cells. Similarly, the effects seen on cells in these micromosaic arrays could also be observed in mosaic microarray immunoassays. However, after careful experimental modulation the issue of assay repeatability and experimental variability can be addressed effectively.

However, the goal is to reduce manufacturing cost, and one viable option is the reusability of the microfluidic array. As for the issue of reusability, the fabrication method of most microfluidic devices mostly required a range of high precision closed in-line devices or stand-alone platforms, which allow new chips to be introduced. This eliminates the ability to interchange sensor surfaces and leaves dispensing cleaning solutions as one of the few alternatives to provide a renewed active surface. This issue has somewhat been addressed and one particularly efficient and flexible system is the use of magnetically captured surfaces as reaction sites [119], which allows an efficient interphasing of microchip device or the use of nanolithography-based surfaces in conjunction with microfluidic network [120]. Currently, there are no efforts that are available to us, where the same array could be used several times except for micromosaic assemblies, which are still in their infancy. However, since antigen-antibody interaction is reversible, therefore, regeneration strategies that are commonly employed in immunobiosensors, such as BIAcore SPR, could be adapted and further tested. 

## 6. Conclusions

We concluded that (i) for clinical diagnostics-based applications focus should be kept at the development of immuno-microfluidic arrays as disposables; (ii) automation and integration of other microfluidic modules should be achieved for real world applications; and (iii) involvement of the industrial partners who are working in the field of diagnostics should be considered exhaustively because the journey of a product from lab to market is essentially facilitated by them. The future and fate of microfluidic microarrays is held by the developments which are holding the full potential of its peripheral technologies, such as conventional microarrays, and are less laborious, simple, and rapid; in our opinion the benefits of microfluidic microarrays as a diagnostic tool outweighs the present limitations.
